# UV imaging reveals facial areas that are prone to skin cancer are disproportionately missed during sunscreen application

**DOI:** 10.1371/journal.pone.0185297

**Published:** 2017-10-02

**Authors:** Harry Pratt, Kareem Hassanin, Lee D. Troughton, Gabriela Czanner, Yalin Zheng, Austin G. McCormick, Kevin J. Hamill

**Affiliations:** 1 Department of Eye and Vision Science, Institute of Ageing and Chronic Disease, University of Liverpool, Liverpool, United Kingdom; 2 Department of Biostatistics, Institute of Translational Medicine, University of Liverpool, Liverpool, United Kingdom; 3 Department of Opthalmology, Aintree University Teaching hospital, Liverpool, United Kingdom; University of Alabama at Birmingham, UNITED STATES

## Abstract

Application of sunscreen is a widely used mechanism for protecting skin from the harmful effects of UV light. However, protection can only be achieved through effective application, and areas that are routinely missed are likely at increased risk of UV damage. Here we sought to determine if specific areas of the face are missed during routine sunscreen application, and whether provision of public health information is sufficient to improve coverage. To investigate this, 57 participants were imaged with a UV sensitive camera before and after sunscreen application: first visit; minimal pre-instruction, second visit; provided with a public health information statement. Images were scored using a custom automated image analysis process designed to identify areas of high UV reflectance, i.e. missed during sunscreen application, and analysed for 5% significance. Analyses revealed eyelid and periorbital regions to be disproportionately missed during routine sunscreen application (median 14% missed in eyelid region vs 7% in rest of face, p<0.01). Provision of health information caused a significant improvement in coverage to eyelid areas in general however, the medial canthal area was still frequently missed. These data reveal that a public health announcement-type intervention could be effective at improving coverage of high risk areas of the face, however high risk areas are likely to remain unprotected therefore other mechanisms of sun protection should be widely promoted such as UV blocking sunglasses.

## Introduction

Despite increasing sun awareness and sun protection usage, between 70–90 percent of basal cell carcinomas (BCCs) develop in sun-exposed head and neck regions, and 5 to 10 percent of all skin cancers occur on the eyelids alone[1]. Specifically within England, 33610 eyelid BCCs were recorded in the 11 years between 2000 and 2010[1]. Within the eyelid area, the medial canthus, a region where the medial corner of the upper and lower eyelids meet, has been shown to be not only a particularly common site for BCC, but is also associated with poor prognosis [2,3,4,5]. It has been postulated that the high prevalence of non-melanoma skin cancer on the eyelids is due to the skin being the thinnest on the body and hence specifically vulnerable to damage from prolonged ultraviolet (UV) light exposure, a well-established risk factor for BCCs and for squamous cell carcinomas [6,7,8,9,10]. Therefore, the importance of adequately protecting this vulnerable area is clear, and the use of sunscreen formulations has been widely promoted.

Use of sunscreens for sun protection requires two conditions to be met: i) adequate quantities of the substance to be applied with appropriate frequency of reapplication, and ii) effective coverage of all sun exposed areas. Importantly, it has been demonstrated that even when the frequency of application and quantity applied are appropriate, the application technique in terms of coverage, is often inadequate [11,12,13,14]. Studies investigating sunscreen application to the face with emphasis on identifying commonly missed areas have very rarely been performed, however, one study in 1994 suggested inferior application to the medial canthal area in a study of 50 participants [15]. In the 23 years since this publication, through numerous public health information streams, awareness of the risks associated with UV exposure has increased dramatically. However, the positive health benefits of UV exposure in terms of supporting the complex sensory functions of the skin, including effects on brain, neuroendocrine, and immune function, as well as the deleterious effects of vitamin D insufficiency have been widely demonstrated leading to guidelines designed to balance the benefits of UV exposure against the potential DNA damage [16,17,18,19,20,21,22,23]. Therefore an updated investigation into application habits is warranted. Recent data suggest that sunscreens are increasingly becoming the method of choice for sun protection meaning that it is more important than ever that sunscreen is applied effectively [24,25,26].

To date, the majority of sunscreen application publications use surrogates in place of real sun creams to determine coverage. Often these surrogates are of different texture or visibility which may influence application [27,28]. Recent improvements in the availability of UV sensitive cameras have opened up the possibility of investigating sunscreen application directly using actual formulations available to the public and obtaining superior image quality compared with the use of a Wood’s lamp [14] or by imaging fluorescent creams [29]. UV photographic imaging has thus been demonstrated as being useful as a method not only to assess sunscreen application but also to assess skin damage and drive behavioral change in sun bed users [30,31,32]. In this study, we have adopted the UV imaging approach to determine if skin cancer prone facial regions are ineffectively covered, and if an information based intervention could be used to improve sunscreen application.

## Materials and methods

### Ethical approval

The study was approved by University of Liverpool Ethics Review Board with approval number 201606181. Written consent was obtained from all participants prior to both phases of the trial. The individuals who’s images have been used in this manuscript have given written informed consent (as outlined in PLOS consent form) to publish their case details here.

### Study design

Sample sizes were determined based on preliminary data where 4 participants were analysed from two identical non-intervention visits and one intervention visit (SD = 8). Power analyses were performed based on 95% power and 5% type I error rate; to detect an increase of at least 5% after intervention requires 57 participants assuming paired t-test. 57 people (27 male and 30 female) were recruited in October to December through poster advertising and an email cascade to all staff and students of the Institute of Ageing and Chronic Disease, University of Liverpool. There were no exclusion criteria based on demographics, ethnicity or other personal criteria, however, excluded from the study were volunteers who self-identified as having allergies to sun lotion. Volunteers were required to fill in a pre-questionnaire before participating in the study (S1 Fig), requiring them to confirm whether they had used any form of sun protection previously, and whether they had any known allergies. Participants were also requested to self-identify their skin-type based on provided Fitzpatrick scale diagram.

Participants were then allowed to self-select from either SPF50 spray or SPF50 cream sunscreen formulations (both Nivea, Birmingham, UK) and instructed to apply sunscreen in their usual manner. No instructions were provided regarding volume of solution to use. UV images were acquired before and after sunscreen application. The same participants were invited to return for a second visit two weeks later, where they received an information sheet stating “Skin cancers most commonly occur on sun exposed areas of the body. Most skin cancers occur in the face with 10% of all skin cancers occurring on the sensitive eyelid area which is thinner and therefore more sensitive to sunlight. Using sunblock reduces the risk of getting skin cancer.” They were then given the same sunscreen formulation as used initially and imaged before and after application. Participants were not shown the images from their first visit prior to sunscreen application. Following the second phase of the study, participants were requested to complete a second questionnaire asking about their experience and to identify behavioural trends within the population (S2 Fig).

### Image acquisition

Participants were sat in front of a plain white background and images captured using a tripod mounted DSLR (Canon EOS Rebel XTi 400D) with 60mm EF-S macro lens (both; Canon, Surrey, UK). The camera was modified to be sensitive only to UV light by replacing the internal hot mirror with a UV band pass filter (Lifepixel, Mukilteo, WA, USA) and were photgraphed with the use of an electronic flash (Vivitar Auto Thyristor Model 285 with fresnel lens removed, Vivitar, Edison, NJ, USA). Camera settings (*F* 2.8, ISO 1800, shutter speed 1.2s,), lighting and distances from the camera were kept constant throughout. The original images were greyscale ten million pixels images in JPG format.

### Image analysis

Manual segmentation was performed using image J (NIH, Bethesda, MA) by an observer manually drawing around areas deemed to be not covered using the freehand selection tool. Due to observers reporting difficulty in identifying glare/reflection and resultant intraobserver variability, this approach was deemed ineffective. Therefore, an automated image analysis method was developed to objectively detect, segment and quantify the areas of the face within the UV images that were not covered by sunscreen. To counteract differences in skin tones and reflection, contrast limited adaptive histogram equalisation was first applied to the images using openCV (http://opencv.org/)[33]. This divided the image into small blocks and each of these blocks were then histogram equalized using the following algorithm:

Let f be the source image and m x n matrix from the small block
The pixel intensity values in a greyscale image vary from 0–255
The probability of an image having intensity x is given by:

$${\text{p}}_{\text{x}}=\frac{\mathrm{pixels}\;\mathrm{with}\;\mathrm{intensity}\;n}{m\;x\;n},\;\text{n}=0,1,\ldots ,255.$$

$${\text{g}}_{\text{m},\text{n}}=255\sum_{\text{x}=0}^{{\text{f}}_{\text{m},\text{n}}}{\text{p}}_{\text{x}}$$

Next, the dlib package (http://dlib.net/) was used to detect facial landmarks within the image. These landmarks were used as markers for determining the facial region and theletterbox region surrounding the eyes of the participant. The landmark located at the inner eye was used to define the medial canthus. The face was segmented from the original image and the relative letterbox points collated for later use. Gaussian blur (openCV code library) was then applied to the normalised image to smooth the image and reduce unwanted noise [34]. The Gaussian equation for a 2D image is defined as:

$$G(x,y)=Ae^{-\frac{{\left(x-u_{x}\right)}^{2}}{2\theta_{x}^{2}}-\frac{{\left(y-\mu_{y}\right)}^{2}}{2\theta_{y}^{2}}}$$

Where μ is the mean pixel value and θ represents the variance.

The image was then mapped to Hue Saturation Value and thresholding performed to produce a binary segmented mask of the image [35]. Results for applying the substance were determined from this binary mask and reported as a percentage of the pixels in each image/letter box region. A binary, yes/no classification was used for the medial canthal region, where images were scored as not covered if the segmentation detected any missed skin within the defined region.

### Statistical approaches

Data were tested for normality with Shapiro Wilk test. Mann-Whitney tests were performed to compare coverage between eyelid regions and non eyelid regions relative to gender, skin type, and sun cream versus sun spray. Spearman’s correlation was used to assess correlation between percentages of eyelid regions missed with percentage of rest of face. Wilcoxon tests were used to assess the improvement in coverage. Chi square test was used to assess change in medial canthal region coverage. Differences were deemed statistically significant where p<0.05.

## Results

### Eyelid regions and medial canthal areas are disproportionately missed during routine sunscreen application

**Fig 1:**
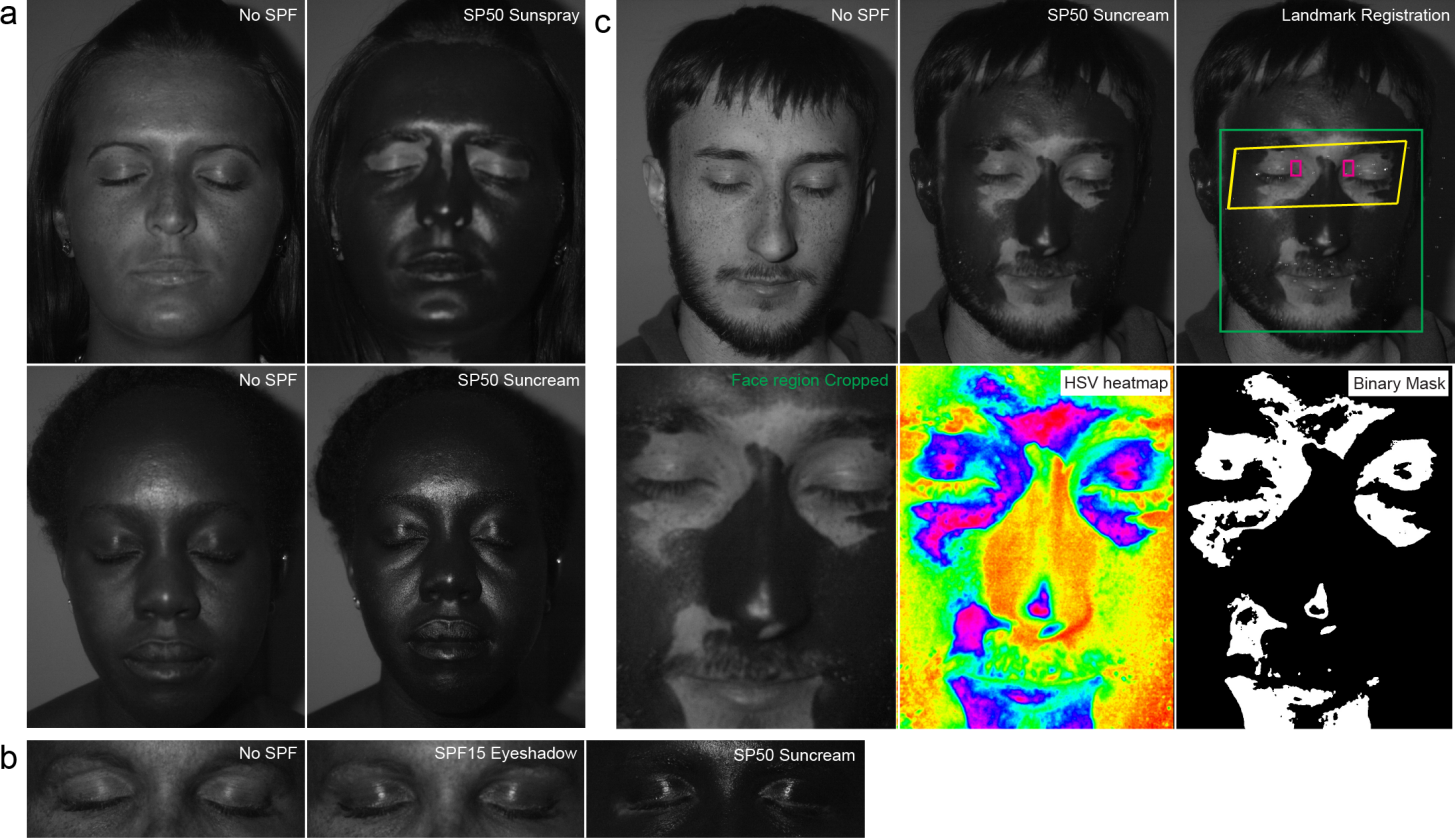
UV imaging as a mechanism to identify regions of incomplete sunscreen application. a) UV images before and after sunscreen application. b) UV images of eyelid region showing the impact of SPF15 makeup compared with SPF50 sun cream. c) UV images analysis steps; top left and middle panels, before and after images from sunscreen application. Top right panel, facial landmarks identified by dlib package: green box; cropped facial region, yellow box; eyelid region, magenta boxes; medial canthal areas. Bottom left, cropped facial region. Bottom middle, hue saturation value (HSV) heat map produced from grayscale image, bottom right binary mask generated from thresholding the HSV heat map.

In order to study how people normally apply sunscreen to their face and thereby identify problem areas, we recruited 57 participants (27 male, 30 female) in October to December 2016 and photographed them using a UV sensitive camera before (Fig 1A) or after (Fig 1B) sunscreen application. UV light is absorbed by melanin and sunscreen, so areas of high pigment or sunscreen coverage appear darker in these photographs whereas non-pigmented skin areas or without sunscreen appear lighter [30]. Analysis of a pre-study questionnaire (S1 Fig) showed all users self-identified as previously having applied sunscreen and nine had applied moisturizer or makeup containing SPF on the day of imaging. No exclusions were required based prior application of SPF nor on skin tone/ethnicity, as fresh application of SPF50 could be detected by the UV camera in all cases (Fig 1A and 1B).

In order to quantify the areas that were/were not covered by sunscreen, we initially attempted to manually segment the images by drawing around the regions using image J software. However, this proved problematic with observers reporting difficulty in differentiating between reflection or glare and true failure in coverage, giving rise to high intraobserver variability in obtained data (S1A Fig). Therefore, in order to remove this subjectivity and account for glare, an automated script was developed. Initially, the images were histogram normalized to counteract differences in skin tone and flash reflection. Facial landmarks were then mapped to the image, and the images cropped (Fig 1C). Next, the areas not covered by sunscreen were segmented through application of a Hue Saturation Value binary thresholding mask (Fig 1C).

Comparison between the manual and automated segmentation revealed that the automated segmentation generally yielded results within the intraobserver variability ranges (S1B Fig), however, the automated segmentation generally identified lower percentage missed in participants where manual observation indicated poor application (S1C Fig), although this difference in scoring did not increase or decrease with type of the skin (S1D Fig). Previously it has been demonstrated that manual segmentation is not only time-consuming, but also produces observer-dependent results, especially for a non-medical study without a clinical definition, which are more reliant on images of uniform light and tone [36,37]. We therefore elected to continue to use the automated system with the understanding that although this may increase risk of underestimation of area missed, it would reduce the risk of obtaining type I errors.

**Fig 2:**
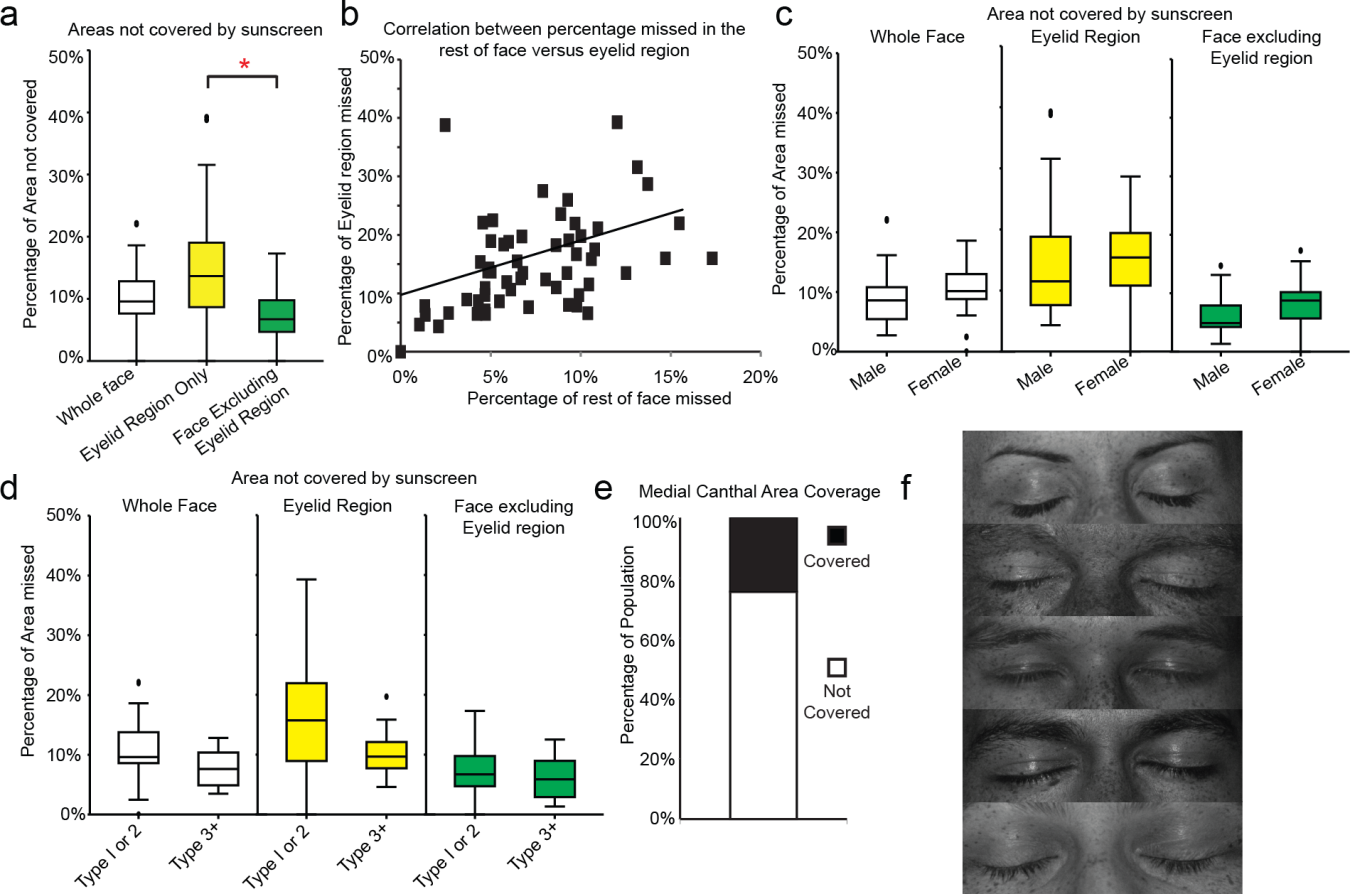
Eyelid regions and medial canthal areas are disproportionately missed during routine sunscreen application. a) Box and whisker plot of percentage of indicated region missed as detected by automated image analysis software. Line represents median, boxes represent 25 to 75^th^ percentile, whiskers 5^th^ and 95^th^ percentile, outliers denoted by black dots, n = 57. * denote significant difference between bracketed groups p<0.01 Mann Whitney test b) Dot plot of percentage area of rest of face missed versus percentage missed in eyelid region. Each dot represents one individual from the trial, n = 57. Spearman correlation coefficient 0.84, p<0.01. c) Box and whisker plots comparing male and females sunscreen application effectiveness plotted as percentage missed of the indicated regions, plotted as in b), d) box and whisker plot comparing application with self-assessed skin type, grouped as types 1 or 2 (n = 42) compared with types 3 or higher (n = 15), plotted as for b), e) Bar chart of percentage of population that either completely covered or failed to cover medial canthal regions. f) Representative UV images of six participants eyelid regions without sunscreen application. Note the dark spots indicating presence of UV damaged skin i.e, areas of pigmentation deep in the dermis that are invisible to the naked eye but visible to UV photography.

Preliminary visual analyses suggested that the eyelid regions were missed with higher frequency compared with the rest of the face. As the eyelid area is particularly prone to skin cancer development [38,39], a letter box region encompassing both eyelids, periorbital regions and the bridge of the nose was isolated from the other facial regions to specifically assess whether this observation reflected a true trend in application behaviour (yellow box in Fig 1C, top panel). Analysis of these data revealed the median percentage of the whole face missed to be 10% (range 0–22%, Fig 2A). Interestingly, a significantly higher percentage of the eyelid region was missed compared with the rest of the face not including the eyelid regions (eyelid median 14%, median non-eyelid 7%, p<0.001 Mann-Whitney test, Fig 2A). Within the cohort, there was a weak positive correlation between the amount of the face not including eyelid missed and the percentage of eyelid region missed (r^2^ = 0.19, Spearman correlation 0.84, p<0.01, Fig 2B) indicating the level of eyelid coverage is more generally related to the overall sunscreen application ability. Comparison between males and females revealed no significant difference between genders (Fig 2C). However, analysis on the basis of self-reported skin type indicated that those with skin types 1 and 2 performed slightly worse than those with skin types 3 or higher, these differences reached significance only for the eyelid regions (eyelid: type 1 or 2 = 16% missed, type 3+ 10%, p<0.05) (Fig 2D). Note the distribution of skin types in this population was skewed toward lighter skin types (n = 42 type 1 or 2, n = 15 type 3+) which is reflective of the local population and therefore these findings should be considered with caution.

As the periorbital/medial canthal regions (magenta box in Fig 1C) are particularly at risk for more aggressive BCC [2,38,39], a secondary binary analysis (covered/not covered) was performed on this region, revealing that 44 of the 57 failed to cover this region (Fig 2E).

In addition to its use for identifying sunscreen application, UV photography also enables the visualization of areas of existing sun damage in lighter complexion individuals. Careful examination of our before sunscreen application images revealed numerous examples of sun damage spots in eyelid regions supporting the concept that this area is at risk for sun damage (Fig 1F).

### Provision of simple risk indication information improved the sunscreen coverage of eyelid regions

Next we sought to determine if increased awareness of eyelid cancer risk would be sufficient to drive an improved coverage of the at risk areas. Various methods of behavioural intervention to promote sun-protection have been previously reported, with varying levels of success. Methods have included written information [32,40], photo aging imaging interactive presentation [41] and psychosocial modelling to assess sun protective behaviour [42]. In this study we chose to use written information as this approach could easily be adopted in the labelling of bottles, moreover studies have shown that awareness of risk is sufficient to drive behavioural changes specifically related to sunscreen use [43].

The same 57 participants were invited to return for a second visit, the study was carried out as previously however prior to sunscreen application participants were provided an information sheet stating; “Skin cancers most commonly occur on sun exposed areas of the body. Most skin cancers occur in the face with 10% of all skin cancers occurring on the sensitive eyelid area which is thinner and therefore more sensitive to sunlight. Using sunblock reduces the risk of getting skin cancer.”

**Fig 3:**
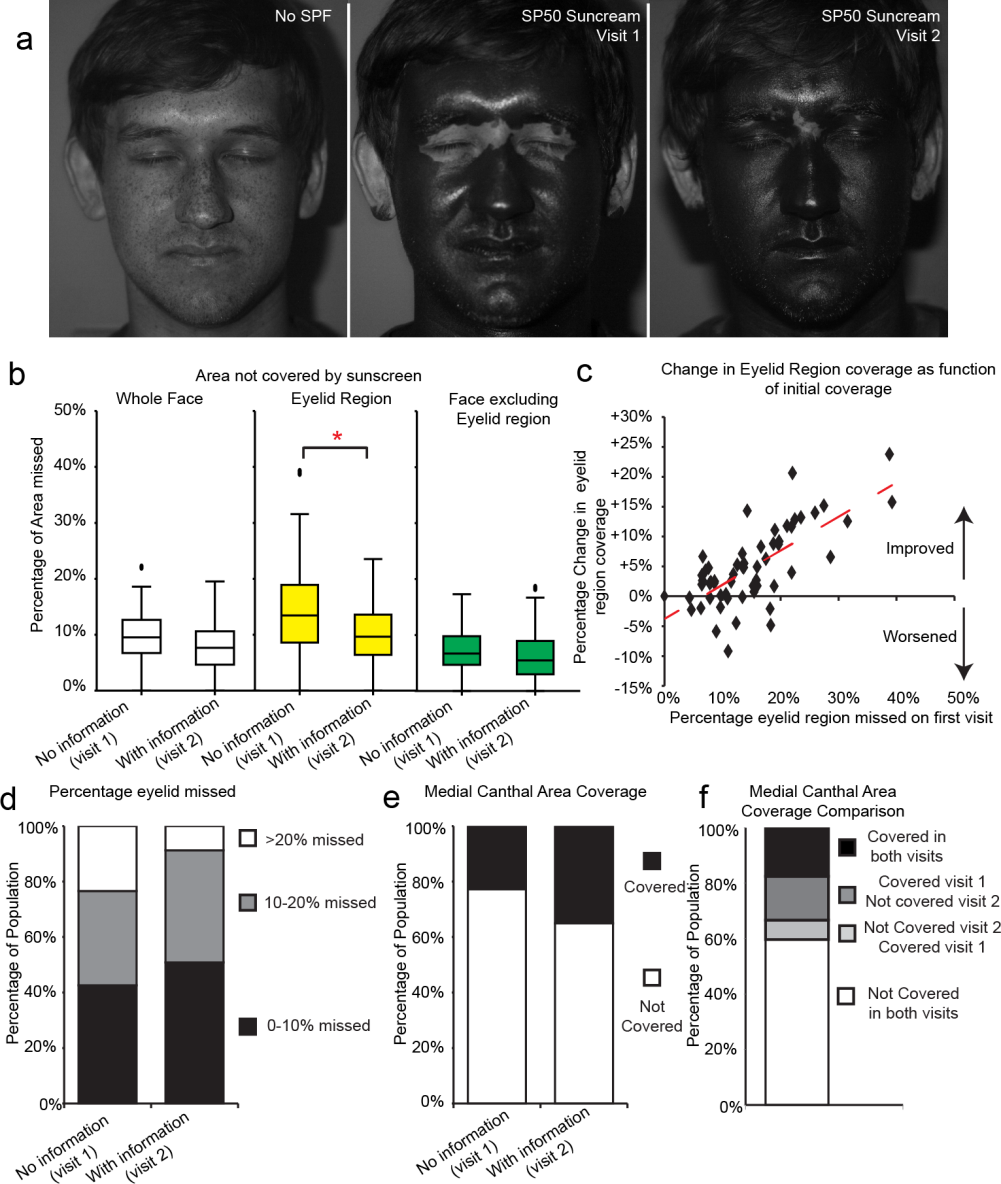
Provision of a simple information sheet improves eyelid coverage. a) Representative images without SPF (left), after SPF50 application during routine application (visit 1), or after receiving cancer risk information sheet (visit 2). b) Box and whisker plot of percentage of region missed as detected by automated image analysis software. Line represents median, boxes represent 25 to 75^th^ percentile, whiskers 5^th^ and 95^th^ percentile, outliers denoted by black dots, n = 57. * denote significant difference between bracketed groups, p<0.01 Wilcoxon Signed Ranks test. c) Dot plot showing percentage coverage change against initial percentage eyelid area missed for all participants (n = 57). Values above 0 on this plot indicate improved coverage. Pearson correlation coefficient 0.66, p<0.01. d) Bar chart showing percentage of study population who failed to cover either >20% of their eyelid regions (white), 10–20% (grey) or 0–10%. e) Bar chart of percentage of population that either completely covered or failed to cover medial canthal regions. f) Bar chart of medial canthal area coverage comparing on an individual basis coverage in visit 1 and visit 2, *x*^2^ p>0.05.

Image analysis revealed at the second visit participants missed a median of 8% of the whole face compared with 10% in the initial visit (Fig 3A and 3B. Range 0–20%, Wilcoxon Signed Ranks test Z -3.63, p<0.001). The percentage of the eyelid region missed in visit 2 showed a statistically significant improvement to a median 10% compared with 14% missed in visit 1 (Fig 3B, eyelid regions from all participants in S4 Fig, range 0–24%, Z -4.66 p<0.001), the non-eyelid regions showed a below significant improvement to 6% missed from 7% (Fig 3B, range 0–19%, Z-1.82, p = 0.069).

Encouragingly, analysis on a per person basis revealed the greatest improvement in eyelid coverage to be to be observed in those who had initially achieved the lowest coverage (Fig 3C, r^2^ = 0.34 Spearman correlation 0.66, p<0.01, Fig 3D, 11/57 missed >20% in visit 1 compared with 5/57 in visit 2). Although statistically significant, the overall eyelid region improvement was relatively small and coverage of the medial canthal area remained poor with 37 of 57 participants still failing to cover this region (Fig 3E and 3F).

**Fig 4:**
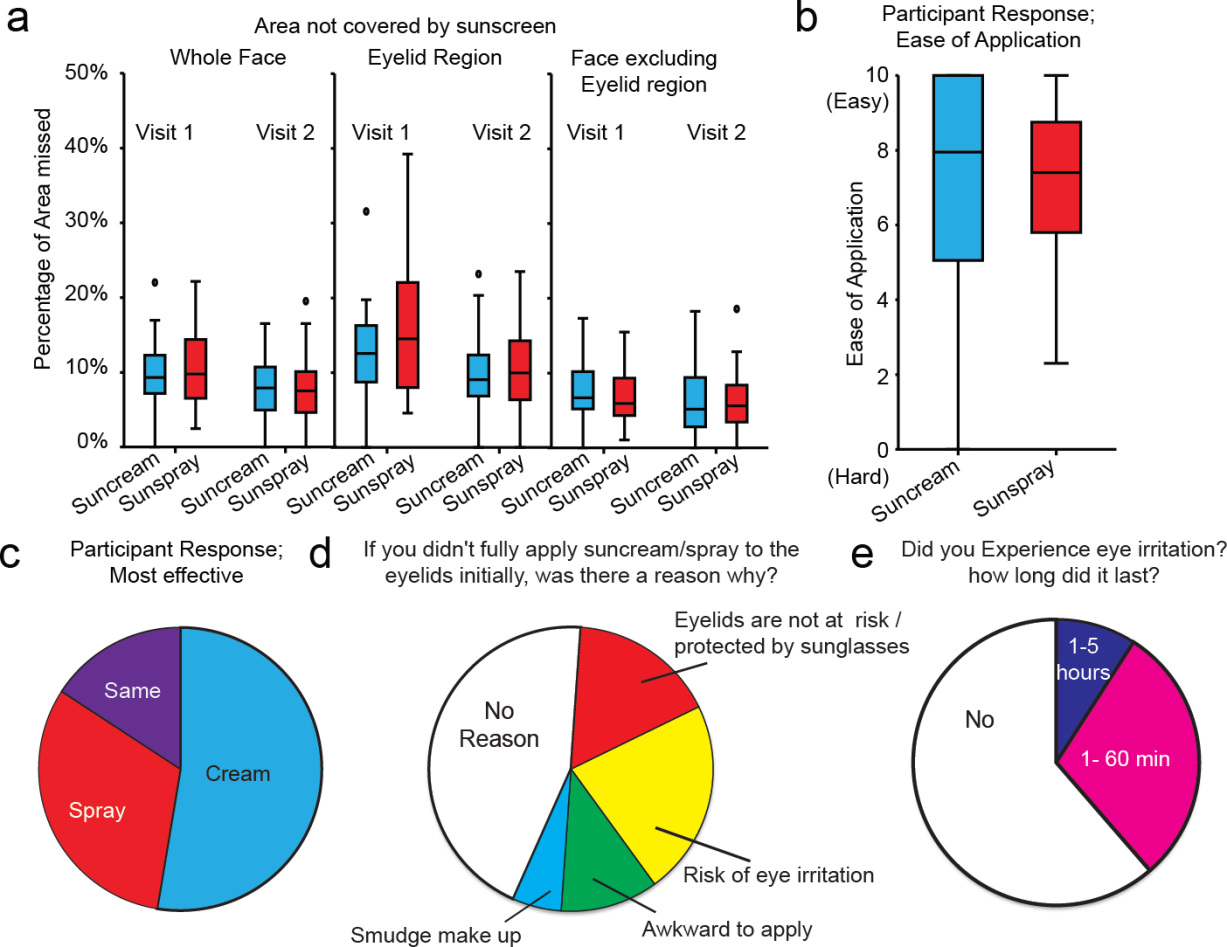
There is no difference between application of sun cream or sun spray. a) Box and whisker plot of percentage of region missed as detected by automated image analysis software. Line represents median, boxes represent 25 to 75^th^ percentile, whiskers 5^th^ and 95^th^ percentile, outliers denoted by black dots, n = 57. p>0.05 Mann Whitney test b) Box and whisker plot of participants Likert scale responses regarding ease of sun cream/spray application. Line represents median, boxes represent 25 to 75^th^ percentile, whiskers 5^th^ and 95^th^ percentile, n = 40. c, d and e) pie charts representing participants' responses to indicated questions.

During the study, participants could choose between use of sun cream or sun spray and were asked to use the same method on both visits. No significant difference was observed in regions missed between users of sun cream and sun spray (Fig 4A, Mann Whitney test). This was unsurprising as all sun spray users first sprayed the solution into their hands and then applied it to their face, as per the manufacturer suggested application method, rather than spraying directly onto the face.

In order to gain an insight into potential reasons why the eyelid regions were missed more frequently, participants were invited to complete a short online questionnaire 1–2 weeks after completing both parts of the study (S2 Fig). Response rate was 78% (44/57). Participants reported approximately equal ease of application (Fig 4B, median score of 80/100 for cream, 73/100 for spray) however, there was a slight perception by the sun cream users of more effective coverage(Fig 4C, 53% Cream, 32% spray, no difference 16%). 20 of 44 respondents answered yes to the question “If you didn't fully apply sun cream/spray to the eyelids initially, was there a reason why?” Stated reasons were; risk of stinging (8/20), no perceived risk/usually rely of sunglasses for eye protection (6/20), awkward to apply to (4/20) and smudging of eye makeup (2/20) (Fig 4B). Consistent with the perceived risk of stinging, 17 respondents reported some eye irritation via the post study questionnaire of which 4 reported “stinging” lasting between 1 and 5 hours (Fig 4E). Finally, 67% of respondents stated that they modified their sunscreen application in response to the provided information sheet.

## Discussion

In this study, we have demonstrated that although overall sunscreen application to the face regularly achieves high levels of coverage, problem areas still exist in the eyelid regions, particularly the medial canthus. However, we demonstrate that provision of a short information sheet is sufficient to drive a small but statistically significantly improved coverage to eyelids regions, which was particularly effective in those individuals that initially performed poorly. These data highlight the need for greater public awareness of eyelid cancer risk and suggest that simple pubic information intervention could be effective. However, our data also demonstrate that, despite improved awareness, the medial canthal areas were still frequently ineffectively covered and therefore use of alternate strategies for protection, such as UV blocking sunglasses, should be promoted wherever possible. This will have the dual effect of protecting cancer at risk areas and also protecting eyes from UV damage, thereby reducing incidence of corneal damage, macular degeneration and cataract formation [44,45].

The participants in our study were drawn from University students and staff members and therefore there is a selection biased toward well-educated participants. Moreover, our participants reflected the local University population and were skewed toward lighter skin types. This has two important implications for the generalizability of our findings. First; one would predict that our cohort would have a base line cancer-risk awareness levels and as such are likely to be vigilant when applying sun protection [43,46]. This may suggest that the disproportionately poor coverage of the eyelid region could be even more striking in the general population. It should be noted, that in our post questionnaire just under half of the respondents did not cite a specific reason for missing the eyelids so, despite a perceived general awareness, their application was still relatively poor. Second; our cohort may respond more to our information based intervention strategy than a broader cross section of the public and therefore we may be over estimating the magnitude of the improvement. However, information based intervention is a standard and proven efficacious approach in wide variety of contexts in diverse groups of patients/participants and as such we do not believe this to be a major limitation here [47,48,49].

When considering the data within this study it is important to consider the behaviour modifying effect wearing sun protection has. If sunscreen application is only performed when preparing for an extended period in the sun, ineffective application will lead to a false sense of security in terms of perceived protection. Our data strongly indicate that in routine application, the eyelid region and particularly the medial canthal areas are relatively poorly protected hence at increased risk. The belief that the whole face is protected may repeatedly increase UV exposure to vulnerable areas that have been missed as people spend longer exposed to the sun.

An important additional consideration is that any public health message must weigh the benefits of reduced UV induced DNA damage against the positive health benefits of UV-B induced vitamin D production. Specifically, insufficient levels of vitamin D have been shown to occur in 50% of the UK adult population with seasonal variations showing severe deficiencies during winter and spring [50,51]. Low vitamin D levels are associated with increased risk of certain cancer subtypes, and other health risks including bone disease, muscle weakness and diabetes mellitus [22,23,52,53,54]. Dietary vitamin D supplementation can reduce some of these health risks [22,53], however, it has also been demonstrated that relatively short UV exposure is sufficient to produce the daily recommended serum vitamin D levels [55]. This is reflected in National Institute for Clinical Excellence (NICE) guidelines which recommend short periods of non-protected exposure to ensure adequate vitamin D production. The findings presented here do not countermand these recommendations, but rather that emphasis be made that, when protection is required, extra attention be paid that sunscreens are applied in a way that does not repeatedly leave the same areas unprotected each time.

Taken together, our findings strongly suggest that a public information campaign is warranted to stress the importance of eye protection from the sun. The ongoing problem of irritation caused by the sun cream/spray needs to be addressed with an appropriate education system put in place to educate the public in newer tear-free formulations [56], whilst the importance of seeking alternate protection mechanisms should be further emphasized.

## Supporting information

S1 Fig Pre study questionnaire. (PDF)

S2 Fig Post study questionnaire. Note that this was presented to participants via an online link.
(PDF)

S3 Fig Comparison between manual and automated segmentation. A. Percentage of cropped eyelid region not covered by sunscreen as determined by manual segmentation. Blue dots represent maximal values without attempting to account for flash reflection/glare. Red dots represent manual segmentation where the observer has attempted to adjust for glare. B. Comparison of automated scoring output (green dots) and manual scoring (red dots) for percentage cover. C. Comparison between mean manual score vs automated score. Dotted line represents exact agreement between scores. D. Differences between manual and automated segmentation scores plotted as divergence from automatic score. For all graphs, data are from 19 images, in A, B, C each vertical column is a single image sorted by self-assessed skin types.
(TIF)

S4 Fig Montage of eyelid region images. Eyelid regions images from all participants showing images taken before sunscreen application (left panels), after sunscreen application in visit 1 (central panels) and after sunscreen application in visit 2(right panels).
(TIF)
