# Supplementary material for: UV imaging reveals facial areas that are prone to skin cancer are disproportionately missed during sunscreen application
Source: PLoS One. 2017 Oct 2;12(10):e0185297. doi: 10.1371/journal.pone.0185297 (PMC5624581; doi:10.1371/journal.pone.0185297)
Supplement: S1 Fig — (PDF) [file pone.0185297.s001.pdf]

Sunscreen Experiment; Pre-trial Questionnaire

Question			
1. Have you ever applied sunscreen before?	YES ☐	NO ☐	
2. Do you prefer using sun cream or sun spray?	Cream ☐	Spray ☐	No Preference ☐

Reason for preference; ....

Question			
3. Do you have any allergies (to sunscreens)	YES ☐	NO ☐	
4. Do you apply face moisturizer?	YES ☐	NO ☐	
4b. If so, do you know whether it contains any sun protective factor?	YES ☐	SPF:	NO SPF ☐
