# Supplementary material for: UV imaging reveals facial areas that are prone to skin cancer are disproportionately missed during sunscreen application
Source: PLoS One. 2017 Oct 2;12(10):e0185297. doi: 10.1371/journal.pone.0185297 (PMC5624581; doi:10.1371/journal.pone.0185297)
Supplement: S2 Fig — Note that this was presented to participants via an online link. (PDF) [file pone.0185297.s002.pdf]

Sunscreen Experiment; Post-trial Questionnaire

1. What did you think of the instructions provided?

0	50	100
Incomprehensible	Adequate	Crystal Clear

Can you please explain your ranking of the instructions

....

2. How did you find the application of the sun tan CREAM to the eyelids?

0	50	100
Impossible	Reasonable	Straightforward

3. How did you find the application of the sun tan SPRAY to the eyelids?

0	50	100
Impossible	Reasonable	Straightforward

4. Which application method did you think was most effective?

Cream ☐
Spray ☐
Both the same ☐ Why? ....

5. When using the sun tan CREAM did you experience any post-application stinging in and around the eyes? If so, for how long?

Yes ☐ How long? ....
No ☐

6. When using the sun tan SPRAY did you experience any post-application stinging in and around the eyes? If so, for how long?

Yes ☐ How long? ....
No ☐

7. Did the change in instructions affect the way in which you applied the sunscreen/spray? Please explain your answer.

Yes ☐ Why? ....
No ☐

8. If you didn’t fully apply suncream/spray to the eyelids initially, was there a reason why? Please explain your answer.

Yes ☐ Why? ....
No ☐
