# Supplementary figures and images for: UV imaging reveals facial areas that are prone to skin cancer are disproportionately missed during sunscreen application
Source: PLoS One. 2017 Oct 2;12(10):e0185297. doi: 10.1371/journal.pone.0185297 (PMC5624581; doi:10.1371/journal.pone.0185297)

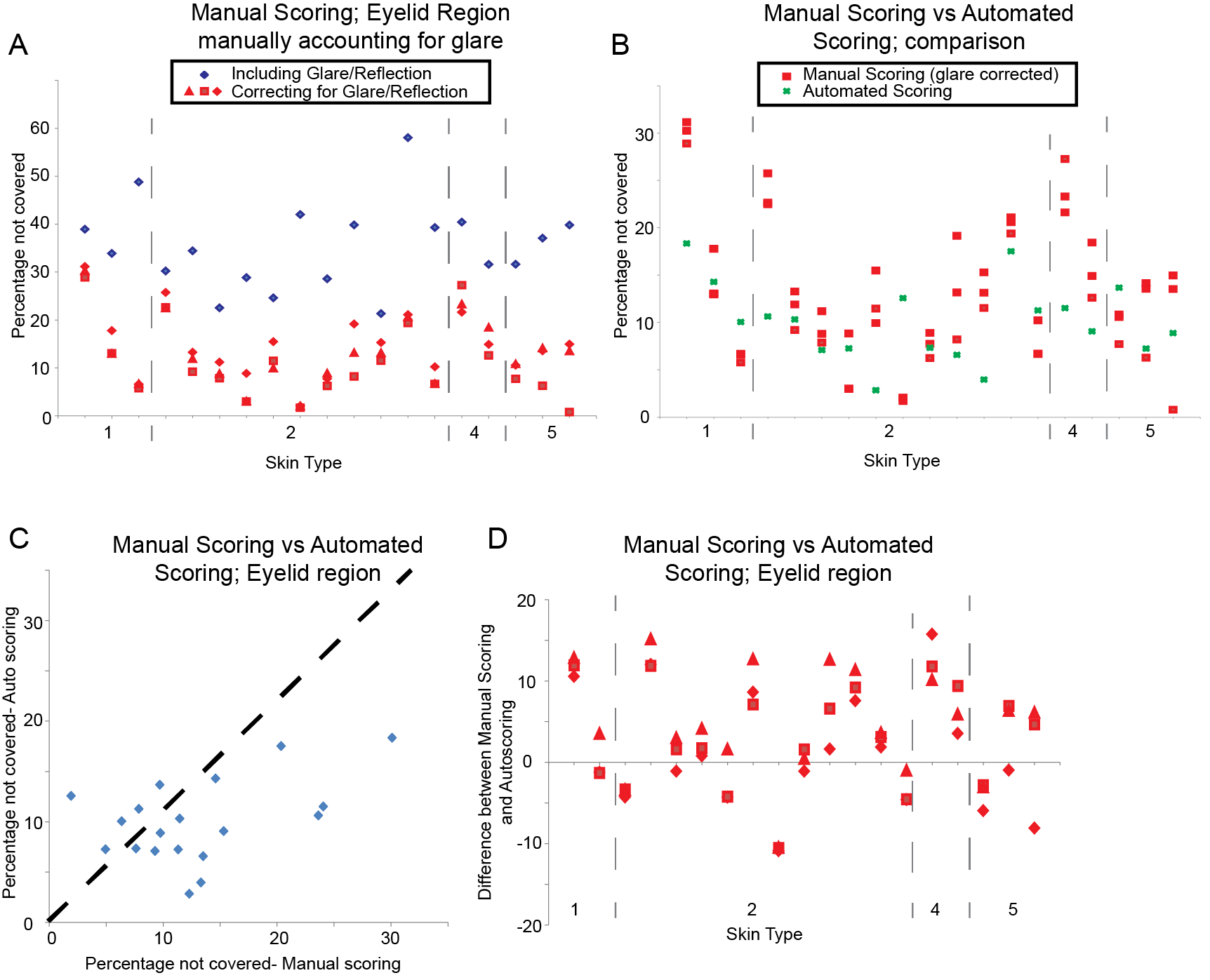

Supplement: S3 Fig — A. Percentage of cropped eyelid region not covered by sunscreen as determined by manual segmentation. Blue dots represent maximal values without attempting to account for flash reflection/glare. Red dots represent manual segmentation where the observer has attempted to adjust for glare. B. Comparison of automated scoring output (green dots) and manual scoring (red dots) for percentage cover. C. Comparison between mean manual score vs automated score. Dotted line represents exact agreement between scores. D. Differences between manual and automated segmentation scores plotted as divergence from automatic score. For all graphs, data are from 19 images, in A, B, C each vertical column is a single image sorted by self-assessed skin types. (TIF) [file pone.0185297.s003.tif]

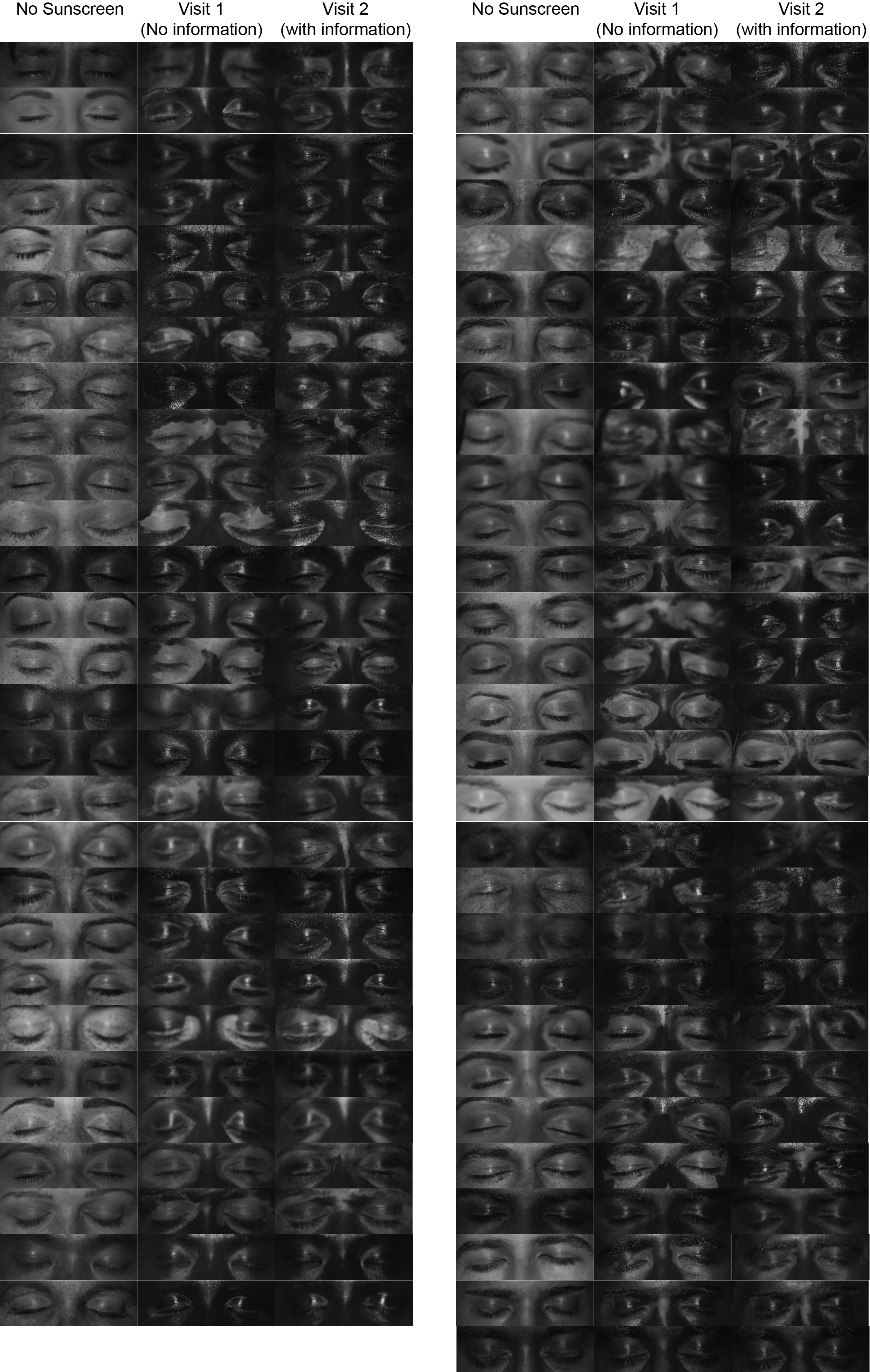

Supplement: S4 Fig — Eyelid regions images from all participants showing images taken before sunscreen application (left panels), after sunscreen application in visit 1 (central panels) and after sunscreen application in visit 2(right panels). (TIF) [file pone.0185297.s004.tif]
